# Out of school female adolescent employment status and sexually transmitted infections (STIs) risk in Uganda: is it a plausible relationship?

**DOI:** 10.1186/s12889-018-6094-1

**Published:** 2018-10-12

**Authors:** Cyprian Misinde, Elizabeth Nansubuga, Olivia Nankinga

**Affiliations:** Department of Population Studies, Makerere University, College of Business and Management Science, School of Statistics and Planning, P.O.Box 7062, Kampala, Uganda

**Keywords:** Adolescent employment, Sexually transmitted infections, Number of lifetime sexual partners, Uganda, Work

## Abstract

**Background:**

High prevalence of Sexually Transmitted Infections (STIs) among adolescents is a serious public health concern. Although many factors are attributed to adolescents’ risk of STI infections, the association between out of school adolescents’ employment status and STIs has not been thoroughly explored in Uganda, yet many adolescents are known to be employed. Consequently, the extent to which adolescents’ employment status may be a risk factor for unprotected sex and STIs among female adolescents is unclear. This study examines the association between female out of school adolescents’ employment status and STI status using the Uganda Demographic and Health Survey (UDHS) data for 2016.

**Methods:**

Both descriptive and multilevel binary logistic regressions were used to analyse the data. The descriptive statistics provided an overview of the association between the two main variables while the multilevel binary logistic estimated the relationship between employment status and STI status, while controlling for other fixed factors and community random factors.

**Results:**

Compared to female adolescents who were not employed or not working, the odds of reporting positive STIs were significantly higher for female adolescents who were working but received no pay (1.801(95% CI = 1.105–2.936), were self-employed in agriculture (1.502 (95% CI = 1.003–2.250) and who did manual jobs (2.258(95% CI = 1.429–3.568) whether skilled or not. Likewise, female adolescents who had two or more lifetime sexual partners (11.679 (95% CI = 1.254–2.248) had significantly higher odds of reporting STIs than those who had only one lifetime sexual partner.

**Conclusion:**

Out of school female adolescents who are employed in various types of low wage employments are at greater risk of exposure to STIs than their counterparts who do not work in Uganda. Instead of being a protective factor against the risk of STIs, their employment may expose them to risky sexual behaviour and STIs, given the vulnerabilities female adolescents who have dropped out of school tend to report. Therefore, the provision of decent employment opportunities for these females may provide an avenue for instructional scaffolding to build the life skills and empower these females so that they are better able to reduce their exposure to STIs.

## Background

Sexually Transmitted Infections (STIs) remain one of the major global sexual and reproductive health problems. Notably, adolescents aged 15–19 years form the second largest group, following 20–24 year olds, with the highest prevalence of STIs [1]. Approximately 20% of adolescents aged 15–19 years reported having STIs or symptoms of STIs in Uganda in 2011 [2]. If not addressed, STIs could lead to serious financial and health costs for both individuals and the wider public [3–5]. Scholars have identified both risk and protective factors for STIs among adolescents; these factors include: adolescents’ sexual behaviours and practices [6], family–parental factors [7], socio-economic factors, and partner characteristics [1]. However the role of adolescent employment status as a potential protective or risk factor for STIs in Uganda has yet to be critically examined, despite the fact that many adolescents who drop out of school are engaged in work. Uganda Bureau of Statistics (UBOS) [8] estimated that approximately 60% of children aged 15–17 years were involved in some form of economic activity. Uganda Bureau of Statistics (UBOS) [8] further estimated that about 21% of females aged 14–17 were engaged in some form of work.

Many studies have assessed the relationship between employment and adolescents’ developmental outcomes such as schooling, grades at school, behaviour such as drug and alcohol consumption and emotional outcomes, but the relationship between employment and STI status has been overlooked. Three major contrasting arguments can be extracted from the literature: (1) there is no relationship between adolescent employment status and developmental outcomes [9–12]; (2) work has a detrimental effect on adolescent behaviour and developmental outcomes [13–17]; (3) adolescent employment correlates with positive developmental outcomes [18]. Moreover, how employment status may affect exposure to sexual behaviours and the potential risk of acquiring STIs for female adolescents, has received little attention. For example, Rich and Kim [16] studied the relationship between adolescents’ current employment status and timing of first sexual encounters and found that employment was associated with an increased likelihood of reporting an earlier first sexual encounter. However, Tenkorang, Rajulton and Maticka-Tyndale [19] found that unemployed youths experienced their first sexual intercourse earlier than their peers who were in employment. The contradictions make it difficult to draw a conclusive argument regarding the role of adolescents’ employment status on involvement in sexual activities and its possible impact on adolescents’ risk of STIs. Evidence exists to show that early sexual encounters are positively related to an increased risk of STIs [20–22]. Evidence further shows that adolescents whose employment requires long hours of work (for example more than 120 h a month) or those who are intensely embedded in work were more likely than non-working adolescents to experience early sexual encounters. Another possible explanation for a link between employment and STIs is that adolescents who are employed may be exposed to other antisocial behaviours, such as drug and alcohol consumption, that could lead to risky sex and STIs [23]. It could also lead to increased association with peers who already exhibit behavioural problems. Mortimer [18] argues that self-selection effects may be evident in this group as adolescents who already exhibit problem behaviours may be more likely to be out of school and engaged in employment rather than their work experience directly contributing to an increased involvement in risky behaviours. Additionally, Mortimer [18] observed that earnings from work may be used to purchase alcohol and drugs. He goes on to argue that when differences in attitudes and behaviours are appropriately taken into account, the negative consequences of employment often disappear.

Adolescents’ sexual behaviours, partner characteristics, parental factors and socio-demographic factors are associated with adolescents STI status. Early sexual encounters have been found to be positively associated with increased odds of reporting of STIs or STI symptoms among adolescents in several studies [20–22, 24]. For example, Kaestle, Halpern, Miller and Ford [21] reported that, STIs were higher among adolescents who experienced their first sexual encounter at age 13 when compared to those whose first sexual encounter was at 17 years.

Cumulative numbers of partners and multiple sexual partners have also been found to be positively associated with STIs among adolescents in many studies [23, 25, 26]. For example, Hahm, Lee, Ozonoff and Amodeo [26] found that sexual experience with more than one partner was significantly associated with positive STI diagnosis. Although, a cumulative number of sexual partners has been found to be strongly associated with the reporting of STIs, some other studies, for example, Santelli, Brener, Lowry, Bhatt and Zabin [23] pointed out that it could be mediated by other factors. However Forhan, Gottlieb, Sternberg, Xu, Datta, McQuillan, Berman and Markowitz [27] found a high prevalence of STIs among adolescents who reported only one lifetime sexual partner. They observed that STIs could be contracted soon after sexual initiation as female adolescents may not have the power to negotiate for safer sex. This is in line with the conclusion of Buffardi, Thomas, Holmes and Manhart [28], that contextual conditions which precede adolescents’ first sexual encounters could increase STI risk by increasing sexual risky behaviours and likelihood of exposure to infections.

Age difference between partners is also strongly associated with STIs among adolescents [25, 29–32]. Gregson, Nyamukapa, Garnett, Manson, Zhuwau et al. [25] found that an older age of sexual partner was associated with increased risk of HIV-1 infection in both men and women but especially among young women whose partners were 5–10 years older. Differences in age of a partner could be mediated by other factors which precipitate the acquisition of STIs. For instance, adolescents are less likely to negotiate safe sex practices, such as condom use [33]. Drumright, Gorbach and Holmes [34] found that STIs were associated with partner’s having multiple sexual partners and ignorance about partner’s sexual behaviour. This study explores the relationship between out of school female adolescents’ employment status and STIs status.

## Methods

The women’s individual recode file from the 2016 Uganda Demographic and Health Survey data (UDHS) was analysed to establish the relationship between female adolescents’ employment status and STI status. In the recode, out of a total of 18,506 women of reproductive age who were interviewed, 1444 adolescents aged 15–19 years reported that they had experienced their first sexual encounter; were sexually active in the year preceding the survey; and were out of school during the year of the survey. This sample of 1444 female adolescents was, selected as the final sample for this study. The sample of out of school female adolescents was selected because it represents a vulnerable population in several ways. This category of adolescents, have often dropped out of school due to poverty and other socio-cultural factors. These female adolescents are often left with no option but to marry at an early age or by force. Due to poverty, most female adolescents are likely to engage in risky sexual behaviours, such as transactional sex, and employment activities for economic survival. Additionally, the power dynamics involved make these adolescents more vulnerable as they have low decision-making power. All these factors pre-dispose them to the risk of STIs. For the overall DHS survey, the sampling was stratified by place of residence and clustered by enumeration areas. Thus, in order to account for the contextual effects of issues surrounding adolescent STIs, enumeration areas were controlled for in the multilevel analyses. There were 577 clusters or enumeration areas. The minimum number of respondents per cluster was one and the maximum was 12. The average number of respondents per cluster was three.

### Variables and measurements

Evidence of a STI is the dependent variable. This variable was constructed from three questions asked to the respondents. The respondents who replied yes to any or a combination of the questions regarding: (1) had a disease acquired through sexual contact; (2) had a bad-smelling abnormal genital discharge; or (3) had a genital sore or ulcer, in the last 12 months, were regarded as reporting a positive STI status. Hence, a binary variable was constructed for this outcome.

The independent variable is employment status derived from two questions: 1) what is your occupation, that is, what kind of work do you mainly do? 2) “Are you paid in cash or kind for this work or are you not paid at all?” Type of employment was categorised based on the International Standard Classification of Occupations (ISCO-08) of 2012. The type of employment was categorised into six classifications, as follows: not working (0); working but receives no pay (1); professional sales and services (2); self-employed in agriculture (3); household/domestic worker (4); and working in manual job whether skilled or not (5). Excluding the not working and those working but receive no pay, all other categories are paid either cash, in kind or both. Due to small sample sizes within categories, analyses were not completed to examine the effects of each mode of payment.

Other variables controlled for in the model included: number of lifetime sexual partners, marital status (never married or ever married), and age difference with recent sexual partner categorised into three groups: 0–4 years, 5–9 years and 10 years and above. Individual characteristics examined in the analyses were age at first sexual encounter, age in years, and education level, while the household factors included wealth status, religion, place of residence, and region of abode. Selection of variables was based on existing research literature and significance at the bivariate level of analysis.

### Statistical analyses

Descriptive statistics and multilevel mixed effects binary logistic regression was used to analyse the data. The descriptive statistics were used to establish the frequency of each variable and a chi square test to establish the simple relationships between the independent and dependent variables. Given that UDHS used multistage stratified sampling procedure, adjustment for complex survey design was done during the computation of the chi square statistics. The mixed effects model was used to establish the association between STI status and female adolescents’ employment status, controlling for other background and household factors as fixed factors; and also controlling for the effects of community factors that could not be examined at fixed level. Stata SE 13 was used for the analyses.

### Ethical consideration

Permission to use the Uganda Demographic and Health Survey data was acquired from the DHS program following the recommended procedure from their website http://dhsprogram.com/data/Access-Instructions.cfm. The data collection process accords rigorous attention to participant’s safety, confidentiality, informed consent and participation in the survey on a voluntary basis. This is done following the World Health Organisation’s ethical and safety recommendations for research [2] and available data contains no information that can be used to identify the respondents.

## Results

Descriptive analyses in Table 1 show that out of 1444 respondents, approximately 20% (*N* = 292) reported a positive STI status in the 12 months preceding the survey. Disaggregation by employment status shows that a third of the respondents (33.4%) were not working, 11.5% worked but received no payment, while 12% of the respondents worked in sales and services and approximately 4% were household or domestic workers.

**Table 1:** Attributes of the respondents

Variables	Frequency	Percent	Variables	Frequency	Percent
STI status in last 12 months			Wealth status		
No	1151	79.77	Poorest	307	21.27
Yes	292	20.25	Poorer	295	20.40
Employment status			Middle	270	18.69
Not employed and not paid	482	33.37	Richer	293	20.30
Employed but not paid	166	11.51	Richest	279	19.34
Sales and services &paid in cash or kind	174	12.07	Religion		
Self- employed in Agriculture and paid in cash or kind	401	27.73	Catholic	450	31.18
Household/domestic worker and paid in cash or kind	60	4.15	Protestant	588	40.74
Manual workers paid in cash or kind	161	11.18	Muslim	204	14.10
Number of lifetime sexual partners			Pentecostal /others	202	13.98
One partner	795	55.02	Place of residence		
Two or more	650	44.98	Urban	316	21.86
Marital status			Rural	1128	78.14
Never married	630	43.7	Region		
Ever married	813	56.4	Kampala	56	3.85
Age difference with recent sexual partner			Central1	187	12.94
0–4	828	57.33	Central2	162	11.24
5–9	449	31.11	Busoga	123	8.51
10 above	167	11.56	Bukedi	139	9.65
Age at first sex			Bugishu	90	6.21
15 and below	654	45.28	Teso	112	7.75
16–19 or in union	790	54.72	Karamoja	22	1.55
Age of respondent			Lango	92	6.37
15–17	554	38.4	Acholi	81	5.61
18–19	889	61.6	West Nile	86	5.95
Education level			Bunyoro	87	6.05
None /Primary	993	68.81	Tooro	101	6.99
Secondary+	450	31.19	Ankole	69	4.79
Total	1444	100.00	Kigezi	37	2.56
			Total	1444	100.00

### Bivariate relationships

Table 2 shows simple relationships between reporting STI status and respondents’ attributes. Disaggregation by employment status revealed that female adolescents who reported doing manual jobs were more likely to report positive STI status than those in other categories of employment or the not-employed group. This relationship was significant (*p* value = 0.025). Other variables that showed significant relationships in bivariate analyses were number of lifetime sexual partners, age of the respondents, wealth status, and region. Based on theory and the relationships observed in bivariate analyses, all these variables were re-analysed in the multilevel logistic regression model.

**Table 2:** Percentage of female adolescents who reported STIs by their attributes in Uganda in 2011

Variables	Percent of women reporting STIs	95% CI	*P* value	Variables	Percent of women reporting STIs	95% CI	***P*** value
Employment status			0.025	Wealth status			0.004
Not Employed and not paid	16.18	12.32,20.97		poorest	14.89	11.13,19.65	
Employed but do not receive any payment	20.07	14.63,26.88		Poorer	19.36	14.93,24.72	
Professional, Sales and services &paid in cash or kind	26.58	18.41,36.74		Middle	19.28	[14.35,25.39]	
Self-employed in agriculture and paid cash or in- kind	18.7	14.93,23.17		Richer	29.35	23.27,36.26	
Household/domestic employee and paid either cash or in-kind	18.69	9.037,34.73		Richest	18.4	13.24,24.98	
Manual labourer and paid cash or in-kind	30.02	22.45,38.86		Religion			0.119
Number of lifetime sexual partners			0.000	Catholic	17.45	13.82,21.79	
One only	15.52	12.78,18.73		Protestant	21.07	17.48,25.17	
two or more	25.99	22.15,30.24		Muslim	17.85	12.13,25.49	
Age difference with current sexual partner			0.131	Pentecostal/others	26.42	20.06,33.95	
0–4	18.02	14.99,21.51		Place of residence			0.234
5–9 years	23.08	18.62,28.24		Urban	23.05	18.06,28.93	
10 years and above	23.55	16.92,31.79		Rural	19.45	16.78,22.43	
Marital status			0.120	Region			0.046
Never married	18.02	14.54,22.12		Kampala	26.28	15.73,40.5	
Ever married	21.95	18.85,25.4		Central1	27.63	19.37,37.76	
Age of first sex			0.521	Central2	17.81	11.08,27.38	
Below 15 years	21.13	17.54,25.23		Busoga	28.45	17.9,42.04	
16–19 or in Union	19.49	16.39,23.02		Bukedi	16.37	10.03,25.58	
Age of respondent			0.010	Bugishu	22.22	15.84,30.24	
15–17	16.21	13.01,20.01		Teso	14.14	9.442,20.65	
18–19	22.74	19.51,26.34		Karamoja	15.63	6.685,32.38	
Highest education level			0.237	Lango	14.67	9.062,22.88	
primary	19.12	16.49,22.07		Acholi	20.89	14.87,28.53	
Secondary+	22.69	17.76,28.52		West Nile	10.34	5.382,18.94	
				Bunyoro	22.84	15.94,31.59	
				Tooro	20.37	13.23,30.02	
				Ankole	25	14.54,39.51	
				Kigezi	8.01	2.957,19.94	

### Odds of reporting STI or the symptoms of among adolescent, by their attributes

Compared to female adolescents who did not work, the odds of reporting STIs increased for all other employment categories, except for household or domestic workers. Compared to female adolescents who did not work, the odds of reporting a positive STI increased significantly for adolescents who: worked but received no pay (1.801 (95% CI = 1.105–2.936); were self-employed in agriculture and received either cash or in-kind payment (1.502(95% CI = 1.003–2.250); as well as those who were doing a manual job (2.258 (95% CI = 1.429,3.568) as shown in Table 3, Model 2. The relationship was significant only amongst adolescents who did a manual job compared to those who were not working in Model 1 Table 3 before controlling for other factors. Even at the bivariate level, female adolescents involved in manual jobs were more likely to report positive STI status than female adolescents in other categories of employment.

**Table 3:** Adjusted odds ratio for female adolescents reported STI status by their attributes controlling for effect of Enumeration area

sti_status	Model 1			Model 2		
	Odds Ratio	*P* value	[95% CI]	Odds Ratio	*P* value	[95% CI]
Employment status						
Not employed ^a^						
Employed in either sales and services, or employee in agriculture but receives no pay	1.377	0.167	0.875, 2.168	1.801	0.018	1.105, 2.936
Professional, Sales and services &paid in cash or kind	1.377	0.176	0.866, 2.189	1.295	0.289	0.803, 2.088
Self-employed in agriculture and paid in cash or kind	1.191	0.347	0.827, 1.715	1.502	0.048	1.003, 2.250
Household/domestic employee and paid either cash or in-kind	0.977	0.954	0.445, 2.149	0.918	0.842	0.397, 2.123
Skilled and unskilled manual worker and paid cash or inkind	1.951	0.003	1.263, 3.012	2.258	0.000	1.429, 3.568
Number of lifetime sexual partners						
one^a^						
two or more				1.679	0.001	1.254, 2.248
Age difference with current sexual partner						
0-4 years^a^						
5–9 years				1.094	0.573	0.801, 1.495
10 years and above				1.324	0.194	0.867, 2.022
Marital status						
Never married^a^						
Ever married				1.045	0.793	0.751, 1.455
Age of first sex						
15 below^a^						
16–19 or in Union				1.050	0.752	0.777, 1.418
Age of respondent						
18–19^a^						
15–17				0.838	0.281	0.608, 1.155
Highest education level						
None/ Primary education^a^						
Secondary+				0.932	0.689	0.658, 1.318
Wealth status						
Poorest^a^						
Poorer				1.490	0.071	0.966, 2.297
Middle				1.386	0.177	0.863, 2.223
Richer				1.951	0.006	1.208, 3.150
Richest				0.952	0.884	0.491, 1.845
Religion						
Catholic^a^						
Protestant				1.251	0.194	0.892, 1.753
Muslim				1.152	0.561	0.715, 1.858
Pentecostal/others				1.606	0.026	1.058, 2.438
STI_Status	Model 1			Model 2		
	Odds Ratio	P value	[95% CI]	Odds Ratio	P value	[95% CI]
Place of residence						
Urban^a^						
Rural				0.936	0.768	0.602, 1.454
Region						
Kampala^a^						
Central1				0.760	0.517	0.331, 1.743
Central2				0.474	0.098	0.196, 1.146
Busoga				1.069	0.879	0.451, 2.532
Bukedi				0.424	0.062	0.172, 1.046
Bugishu				0.496	0.141	0.195, 1.261
Teso				0.350	0.030	0.135, 0.902
Karamoja				0.397	0.143	0.115, 1.368
Lango				0.422	0.079	0.161, 1.107
Acholi				0.635	0.349	0.246, 1.641
West Nile				0.290	0.019	0.103, 0.816
Bunyoro				0.675	0.404	0.269, 1.699
Tooro				0.600	0.272	0.241, 1.492
Ankole				0.698	0.467	0.265, 1.837
Kigezi				0.195	0.014	0.053, 0.715
_cons	0.184	0.000	0.138, 0.246	0.160	0.000	0.058, 0.443
Random effects						
	Estimates	Std. Err.		Estimates	Std. Err.	
EA	0.242	0.196	0.050, 1.179	0.031	0.167	0.000, 1339.132

Model 1: LR test vs. logistic regression: chibar2(01) = 1.97 Prob> = chibar2 = 0.0805

Model 2: LR test vs. logistic regression: chibar2(01) = 0.03 Prob> = chibar2 = 0.4260

*CI* Confidence Interval, *EA* Enumeration Area

^a^reference category

Number of lifetime sexual partners was significantly associated with female adolescents’ STI status. The odds ratio of reporting STI was higher among female adolescents who reported having had two or more lifetime sexual partners compared to those who reported having only one lifetime sexual partner. Likewise, among the regions, the odds of reporting STIs decreased significantly for adolescents who lived in Teso, West Nile and Kigezi compared to Kampala region.

## Discussion

This study’s objective was to understand the association between out of school female adolescents’ employment status and STI status in Uganda, controlling for female adolescents’ sexual behaviours and background characteristics. The results suggested that STI prevalence among out of school female adolescents was higher among the employed group than adolescents who did not work, excluding adolescents who worked as households/domestic employees, when the confounding factors were taken into account. The higher odds of reporting positive STIs were significant for female adolescents who worked but received no pay, were self-employed in agriculture, and for those who were engaged in manual work; which is in congruence with the study conducted by Rich and Kim [16]. However, this finding is in stark contradiction with other studies which highlight adolescent employment as a protective factor against STIs and risky sexual behaviours [19]. Without undermining the value of female adolescent employment [18], the increasing odds of reporting STIs among these female adolescents could be due to the heightened vulnerability of these groups of female adolescents. Their vulnerability may influence school dropout during adolescence and therefore weaken the protection against risky sexual behaviours and STIs schools provide to adolescent girls [35]. Yet adolescents’ psycho-social needs for a modern lifestyle (e.g. smart phone and data), may not be easily met by the jobs they do; this could increase their vulnerability to transactional sex and exposure to STIs [36]. Adolescents who work but do not receive pay, in addition to those who are self-employed in agriculture and in manual jobs receive little to no pay [37] which increases the vulnerability of these female adolescents. The low paying jobs could also expose the female adolescents to sexual exploitation that could increase the risk of STIs [38]. Thus the high level of vulnerability of out of school female adolescents who are in different types of employment exposes them to the risk of unsafe sex and STIs which is in accordance with other studies [39, 40]. This is perhaps because these female adolescents cannot negotiate for safer sex, hence an increased exposure to the risk of STIs [27]. The female adolescents who do not work could face similar vulnerability, however, the reduced risk of STIs could be attributed to the gains from parental monitoring [39], which is not possible for adolescents who may be working outside the home or away from parents. An alternative explanation could be, that adolescents who already have developmental problems may use work to gain access to behaviours that would be confined to only adults, including sexual behaviours and drug use, increasing exposure to peer groups that may lead them to engage in risky sex and STIs [36, 39]. Mortimer [18] argues that this could occur as a result of self-selection, however, the fact that adolescents who worked without receiving renumeration also reported increased odds of STIs suggests that their vulnerability over and above self-selection effects may be the reason for this relationship.

Apart from female adolescent employment status, number of lifetime sexual partners was an additional significant factor associated with female adolescents’ positive STI status. STIs were more prevalent among female adolescents who reported having had more than one lifetime sexual partner compared to those who had only one lifetime sexual partner, which is in agreement with other studies that stress that the cumulative number of sexual lifetime partners is a risky factor for adolescents STI transmission [23, 25, 26]. Likewise, the odds of reporting STIs were significantly higher among adolescents affiliated to the Pentecostal religion than for Catholics. This perhaps can be attributed to the fact that some people, including adolescents, join the Pentecostal religion in a quest for deliverance from the problems they face. The problems may include health problems, such as STIs/HIV, or behavioural problems which may include risky sexual behaviour. In contrast, the odds of reporting STIs were significantly lower among adolescents who lived in Teso, West Nile and Kigezi regions than in Kampala. Perhaps this is due to cultural reasons such as strong norms about sexual relationships for female adolescents or high level of exposure in Kampala which could lead the female adolescents to initiate sex early, hence increasing their exposure to STIs.

### Strengths and limitations of this study

One of the strengths of this study is the use of the most recent UDHS 2016 data set. These data is not only nationally representative but also provides the most up-to-date and comprehensive information about adolescents’ STI status in Uganda. Secondly, the multilevel logistic regression is a robust method that takes into account the effects of enumeration areas on reporting STI among female adolescents. However, there are also some study limitations. STI-status (the dependent variable), is self-reported; this is associated with the limitations of both under and over reporting of STIs. However, the data can be used to inform us of the overall picture of female adolescent STI-status in Uganda. Another limitation of this study is that, the data comes from a cross-sectional survey, which has a number of limitations, for example recall bias due to limited memory. Moreover, employment categories are not exhaustive, for example information about sex work which is a high-risk activity for STI transmission is not collected, since it is deemed an illegal activity. Future studies may focus on undertaking qualitative studies to explore the in-depth factors associated with STI risk among out-of-school female adolescents.

## Conclusion

Out-of-school female adolescents who are employed in various types of low wage employments are at greater risk of exposure to STIs than their counterparts who do not work. Instead of being a protective factor against the risk of STIs, their employment could be a means of exposure to risky sexual behaviour and STIs given their vulnerability. These adolescents lack skills and are not empowered, hence may not be able to negotiate for improved wages or better working conditions. In the similar vein, they may not be in a position to negotiate for safe sex which exposes them to the risk of STIs.

This study recommends that opportunities for decent work should be provided to out of school female adolescents, which could enhance their acquisition of life skills, such as a sense of judgement, problem solving skills, competencies, self-worth and productive wages. Any employment opportunity that enhances female adolescents’ ability to acquire life skills, at the same time as providing a productive wage, could protect female adolescents against risky sexual encounters which may increase their exposure to STIs including HIV. Therefore, the provision of decent employment opportunities should be an avenue for instructional scaffolding for out of school female adolescents to build their life skills and become empowered. The areas for scaffolding should perhaps include resources and issues concerning reproductive health knowledge and practices among others. For adolescents who are engaged in employment and earn some income, they should be empowered in terms of knowing how best to spend their income in profitable investments rather than spending it on detrimental behaviours that could expose them to the risk of STIs.

## Abbreviations

CI: Confidence Interval
DHS: Demographic and Health Surveys
HIV: Human immunodeficiency virus
STI(s): Sexually Transmitted Infections
UBOS: Uganda Bureau of Statistics
UDHS: Uganda Demographic and Health Surveys
